# Brassinin Induces H_2_S Signals and Improves Vascular Smooth Muscle Cell Functions

**DOI:** 10.3390/molecules30183775

**Published:** 2025-09-17

**Authors:** Jazmin Fergani, Xiaoli Han, Zhuping Jin, Yanxi Pei, Sabine Montaut, Guangdong Yang

**Affiliations:** 1 School of Natural Sciences, Laurentian University, Sudbury, ON P3E 2C6, Canada; jfergani@laurentian.ca; 2 Cardiovascular and Metabolic Research Unit, Laurentian University, Sudbury, ON P3E 2C6, Canada; 3 School of Life Sciences, Shanxi University, Taiyuan 030006, China; 202323101009@email.sxu.edu.cn (X.H.); jinzhuping@sxu.edu.cn (Z.J.); peiyanxi@sxu.edu.cn (Y.P.)

**Keywords:** H_2_S, brassinin, vascular smooth muscle cells, angiotensin II, oxidative stress, inflammation

## Abstract

Brassinin, a sulfur-containing phytoalexin, exerts anticancer and anti-inflammatory effects. Hydrogen sulfide (H_2_S) is an important gasotransmitter with significant cardioprotective properties. The effects of brassinin on H_2_S signaling and vascular smooth muscle cell (SMC) functions remain unexplored. This study found that brassinin protected against angiotensin II (Ang II)-induced SMC dysfunctions. These effects included the attenuation of excessive cell proliferation, migration, and oxidative stress; and upregulation of smooth muscle contractile protein expressions; and down-regulation of inflammatory gene expressions. Notably, brassinin did not directly release H_2_S under the tested conditions; instead, it stimulated endogenous H_2_S synthesis in cultured SMCs by inducing the expression of cystathionine gamma-lyase (CSE), a key H_2_S-generating enzyme. Further mechanistic investigations revealed that brassinin may bind to the transcription factor C/EBPβ and enhance its interaction with the CSE promoter, thereby upregulating CSE transcription. In conclusion, our findings demonstrate that brassinin protects against SMC dysfunction, at least in part, by activating H_2_S signaling rather than acting as a direct H_2_S donor. These results provide new insights into the potential of brassinin as a therapeutic agent for improving vascular health and preventing cardiovascular diseases.

## 1. Introduction

Cardiovascular diseases (CVDs) are the leading cause of death globally, responsible for approximately 1 in every 5 deaths worldwide. A major driver of CVD development and progression is vascular smooth muscle cell (SMC) dysfunction [1]. In the circulatory system, SMCs play a critical role in maintaining vascular health by regulating blood pressure and adapting vascular structure in response to various physiological cues [2]. However, under stressful conditions—such as hypertension, oxidative stress, or inflammation—vascular remodeling can occur, triggering SMC dysfunction [3]. These functional impairments are characterized by the downregulation of contractile smooth muscle genes, alongside increased cell proliferation and migration [2,3]. A key inducer of SMC dysfunction is angiotensin II (Ang II), a major peptide hormone produced by the renin-angiotensin system (RAS) [4]. As a potent vasoconstrictor, excessive Ang II production promotes vascular remodeling and sustained vasoconstriction, primarily through binding to the Ang II receptors on SMCs [5,6].

Hydrogen sulfide (H_2_S), an important gasotransmitter, regulates a broad range of physiological functions, with particular relevance to the cardiovascular system through its antioxidant and anti-inflammatory actions [7,8]. H_2_S can be generated in the body via both endogenous and exogenous pathways. In the cardiovascular system, the majority of endogenous H_2_S synthesis is mediated by cystathionine gamma-lyase (CSE), which catalyzes the metabolism of L-cysteine using pyridoxal 5′-phosphate (P5P) as a cofactor [9]. 3-mercaptopyruvate sulfurtransferase (3-MST) has also been reported to produce H_2_S in vascular system [7]. Additionally, H_2_S can be derived from sulfur-containing amino acids and natural sulfur compounds through non-enzymatic pathways [10,11]. H_2_S is critical for maintaining normal SMC functions, including promoting vasodilation and inhibiting proliferation, migration, and calcification, among other processes [2,12]. A lower level of H_2_S has been linked to various CVDs, such as hypertension, atherosclerosis, and heart disease [13,14].

Numerous epidemiological studies have demonstrated an inverse association between increased consumption of green vegetables—particularly cruciferous varieties—and a reduced CVD risk [15,16]. Many natural sulfur-containing compounds derived from vegetables exert beneficial effects on cardiovascular health by releasing H_2_S under various physiological conditions [17,18]. Brassinin, a sulfur-containing phytoalexin produced de novo in plants of the Brassicaceae family in response to biotic or abiotic attack, has garnered significant research interest due to its health-promoting properties. Brassinin exhibits anticancer effects by inducing phase II drug-metabolizing enzymes, act as an anti-inflammatory agent by inhibiting NF-κB transactivation involved in inflammatory processes, and function as an antioxidant [19,20,21,22]. Additionally, brassinin has been identified as a potential inhibitor of tyrosinase and melanogenesis [23]. However, despite these advances, the potential role of brassinin as an H_2_S donor and its protective roles in regulating SMC functions remain unclear.

The present study aimed to investigate the regulatory effects of brassinin on SMC functions, with a specific focus on its influence on H_2_S signaling under various conditions. Our results demonstrate that brassinin protects against Ang II-induced SMC phenotypic switching. Additionally, brassinin induces H_2_S synthesis through C/EBPβ-mediated transcription of CSE.

## 2. Results

### 2.1. Brassinin Inhibits Ang II-Induced SMC Phenotypic Changes

**Figure 1:**
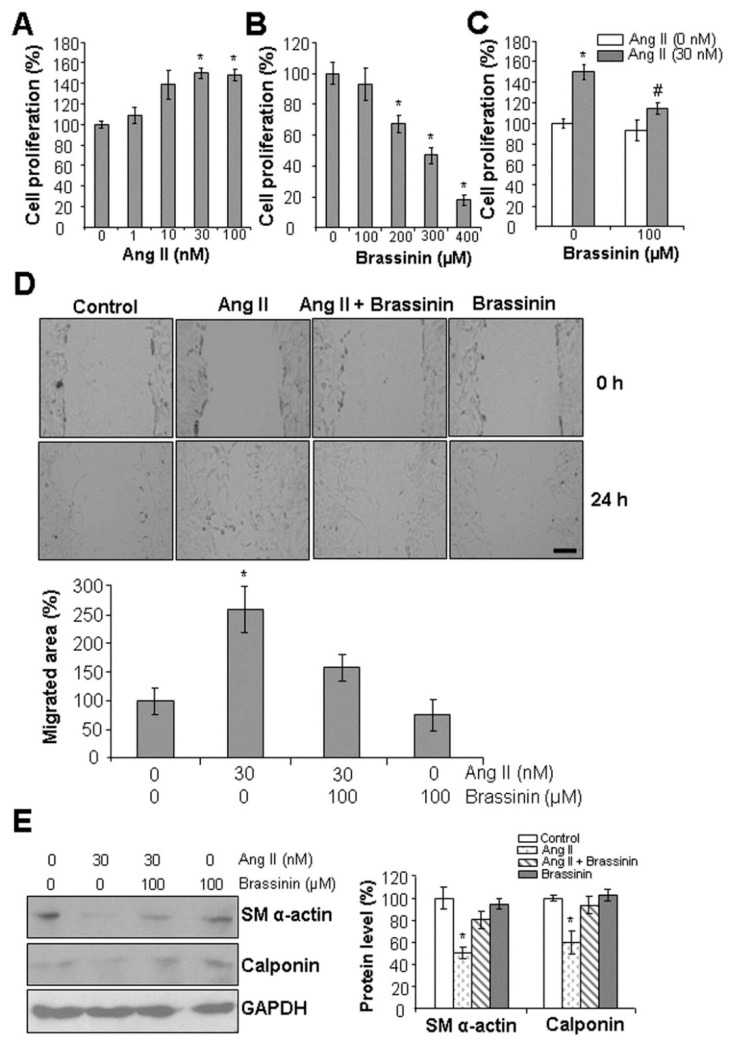
Brassinin protects from Ang II-induced SMC phenotypic changes. (**A**), Ang II at 30 nM started to significantly induce SMC proliferation. (**B**), Brassinin at 100 µM had no effect on SMC proliferation. (**C**), Brassinin suppressed Ang II-induced SMC proliferation. In (**A**–**C**), after SMCs were incubated with varying concentrations of Ang II in the presence or absence of brassinin for 24 h, the cells were subjected to MTT assay. n = 3. *, *p* < 0.05 versus the control cells without any treatment; #, *p* < 0.05 versus the cells treated with Ang II only. (**D**), Brassinin attenuated Ang II-induced SMC migration. Cell migration was assessed with wound healing assay during the 24 h-incubation time with 30 nM Ang II and/or 100 µM brassinin. The cell images were taken with an EVOS M5000 fluorescent microscope at 0 h and 24 h after the cells were scratched with 100 µL tips. Scale bar: 100 µm. *, *p* < 0.05 versus all other groups. (**E**), Brassinin reversed Ang II-reduced expressions of SM α-actin and calponin. After SMCs were incubated with 30 nM Ang II and/or 100 µM brassinin for 24 h, the cells were collected for Western blotting analysis of protein expressions. n = 3. *, *p* < 0.05 versus all other groups.

Vascular SMCs are primarily characterized by their contractile function, accompanied by low proliferation and migration rates [2,3]. Under stressful conditions, SMCs can undergo a phenotypic transition from a contractile to a synthetic, proliferative state, contributing to vascular remodeling and the development of vascular disorders [1]. Ang II is known to induce SMC phenotypic change [5,6]. As shown in Figure 1A, 30 nM Ang II significantly induced SMC proliferation; however, increasing the Ang II concentration to 100 nM did not further alter proliferation. Figure 1B demonstrated that 100 µM brassinin had no effect on SMC proliferation, whereas brassinin concentrations above 100 µM reduced cell proliferation. Notably, 100 µM brassinin significantly reversed SMC proliferation stimulated by 30 nM Ang II (Figure 1C). To further assess brassinin’s anti-migratory properties, a wound healing assay was conducted. As depicted in Figure 1D, Ang II treatment increased SMC migration by approximately 2.6-fold compared to the control group. In contrast, 100 µM brassinin significantly reversed this Ang II-stimulated migration. Importantly, brassinin alone had no impact on basal cell migration. Additionally, Ang II significantly downregulated the protein expression of smooth muscle α-actin (SM α-actin) and calponin—two key markers of the contractile SMC phenotype. This reduction was restored by co-incubation with brassinin (Figure 1E). Collectively, these findings demonstrate that brassinin protects SMCs from Ang II-induced phenotypic switching.

### 2.2. Brassinin Attenuates Ang II-Induced Oxidative Stress and Inflammatory Gene Expressions

**Figure 2:**
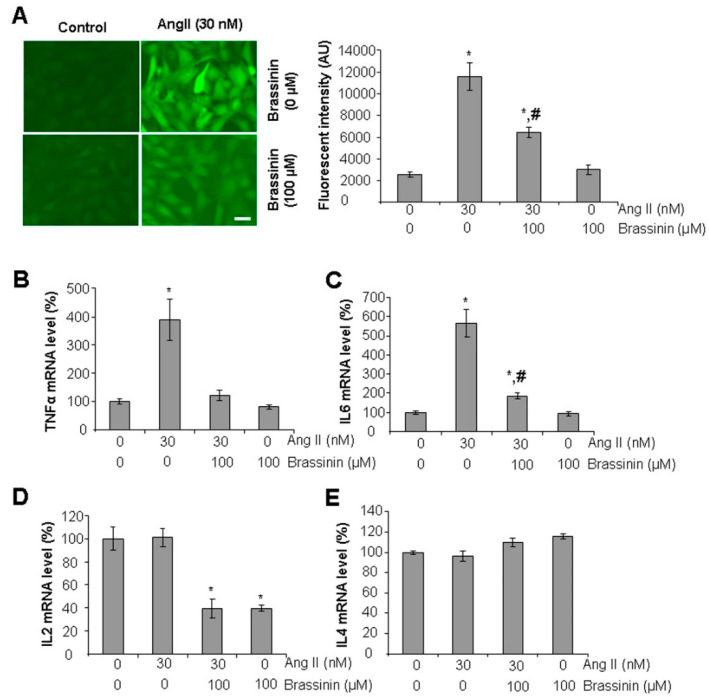
Brassinin attenuates Ang II-induced oxidative stress and inflammatory gene expressions. (**A**), Brassinin inhibited Ang II-induced ROS level. After SMCs were incubated with 30 nM Ang II in the presence or absence of 100 µM brassinin for 24 h, the cells were subjected for measuring oxidative stress by staining with H2DCFDA. The fluorescent intensity was quantified with Image J software. AU, arbitrary unit. Scale bar: 20 µm. *, *p* < 0.05 versus the control group; #, *p* < 0.05 versus the group treated with Ang II only. (**B**–**E**), Brassinin suppressed Ang II-induced inflammatory gene expressions. After SMCs were incubated with 30 nM Ang II in the presence or absence of 100 µM brassinin for 24 h, the cells were collected for real-time PCR analysis of TNFα (**B**), IL6 (**C**), IL2 (**D**), and IL4 (**E**). n = 3. *, *p* < 0.05 versus the control; #, *p* < 0.05 versus the group treated with Ang II only.

To assess the antioxidant effects of brassinin, we used the fluorescent probe H_2_DCFDA to measure reactive oxygen species (ROS) levels in SMCs. As shown in Figure 2A, Ang II-treated cells displayed a significant increase in fluorescence intensity in comparison with the control cells. Co-treatment with 100 µM brassinin markedly reduced the fluorescent intensity brough up by Ang II, indicating that brassinin effectively attenuates Ang II-induced oxidative stress. Importantly, brassinin alone had no impact on basal oxidative stress levels. To further characterize the anti-inflammatory properties of brassinin in cultured SMCs, we analyzed the expression of inflammatory genes using real-time PCR. Ang II treatment significantly upregulated TNFα and IL6 mRNA levels, but these increases were attenuated when cells were co-incubated with brassinin (Figure 2B,C). Additionally, brassinin suppressed IL2 mRNA levels both in the presence and absence of Ang II (Figure 2D). In contrast, neither Ang II nor brassinin had any effect on IL4 mRNA expression (Figure 2E).

### 2.3. Brassinin Inhibited Ang II-Induced AT1R Gene Expression

**Figure 3:**
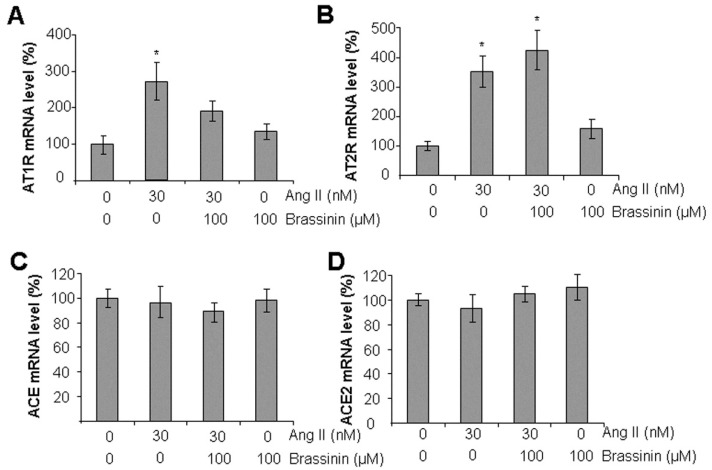
Brassinin suppresses Ang II-induced AT1R mRNA expression. After SMCs were incubated with 30 nM Ang II in the presence or absence of 100 µM brassinin for 24 h, the cells were collected for real-time PCR analysis of AT1R (**A**), AT2R (**B**), ACE (**C**), and ACE2 (**D**). n = 3. *, *p* < 0.05 versus the control.

To investigate the mechanism by which brassinin protects against Ang II-induced disruption of SMC functions, we analyzed the mRNA levels of the two major Ang II receptors—AT1R and AT2R—using real-time PCR. Our results showed that Ang II significantly upregulated both AT1R and AT2R mRNA expression. However, brassinin supplementation specifically reversed the Ang II-induced increase in AT1R mRNA levels, with no significant effect on AT2R expression (Figure 3A,B). Additionally, neither Ang II nor brassinin altered the mRNA expression of ACE or ACE2—two key enzymes involved in Ang II generation and metabolism (Figure 3C,D). These findings suggest that brassinin may protect against Ang II-induced SMC phenotypic switching by targeting AT1R.

### 2.4. Brassinin Does Not Release H_2_S Naturally

**Figure 4:**
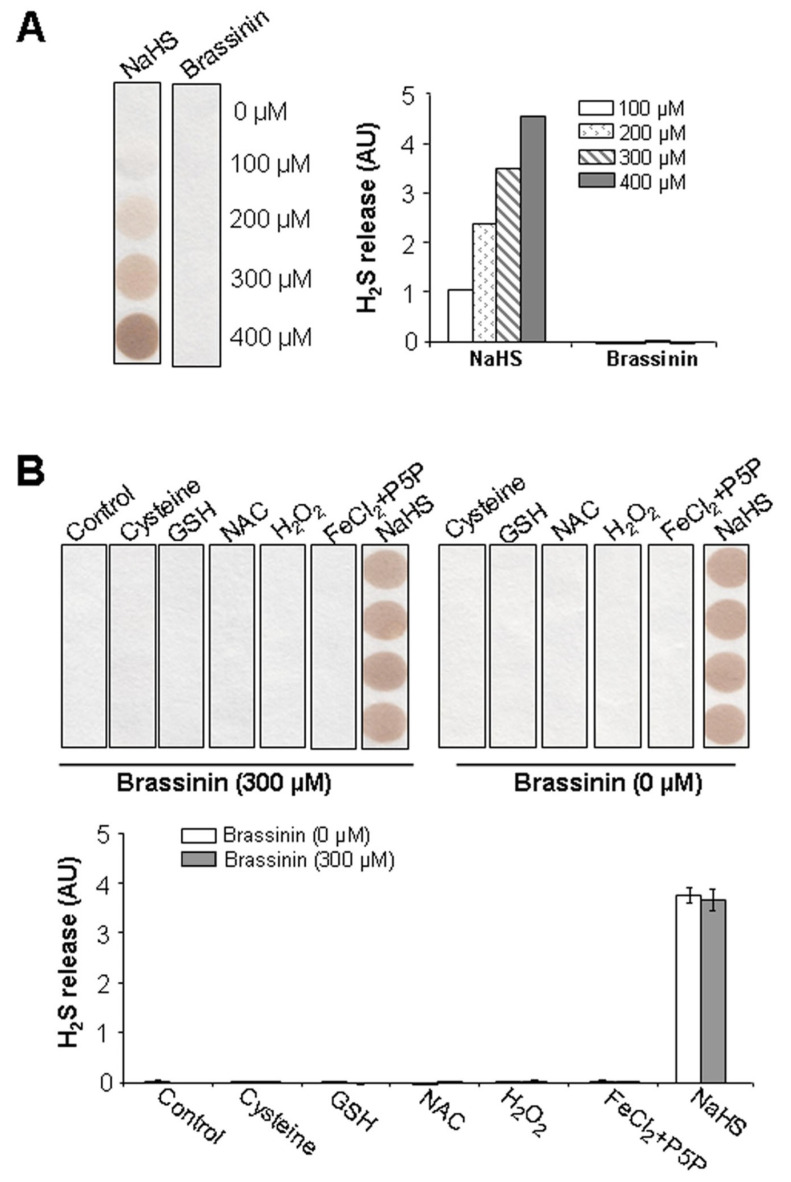
Brassinin does not release H_2_S naturally. (**A**), NaHS and brassinin at 100–400 μM were prepared for H_2_S release measurement at 37 °C for 2 h with lead acetate paper and quantification using Image J software. AU, arbitrary unit. (**B**), Supplement with L-cysteine, GSH, NAC, H_2_O_2_, FeCl_2_ and P5P, or NaHS did not liberate H_2_S release from brassinin. Brassinin at 300 μM was mixed with 10 mM L-cysteine, 10 mM GSH, 10 mM NAC, 100 μM H_2_O_2_, 100 μΜ FeCl_2_ and 1 mM P5P, or 300 μM NaHS, then incubated at 37 °C for 2 h. H_2_S release was trapped by lead acetate paper and quantified using Image J software. n = 4.

Brassinin is a sulfur-containing phytoalexin [22]. To determine whether it can naturally release H_2_S, we prepared brassinin at various concentrations (0–400 μM) and assessed H_2_S release using lead acetate paper. This method visually detects H_2_S through the formation of a brown lead sulfide precipitate. As shown in Figure 4A, the positive control (NaHS, 0–400 μM) released H_2_S in a dose-dependent manner, as confirmed by the progressive formation of the lead sulfide precipitate. In contrast, no precipitate was observed for any concentration of brassinin, indicating that brassinin does not naturally release H_2_S.

To further investigate whether brassinin releases H_2_S under oxidative or reductive conditions, 300 μM brassinin was incubated with various agents: 10 mM L-cysteine, 10 mM glutathione (GSH), 10 mM N-acetylcysteine (NAC), 100 μM H_2_O_2_, or a combination of 100 μM FeCl_2_ and 1 mM P5P. As shown in Figure 4B, no visible lead sulfide precipitate was formed in any of these co-incubation conditions. These results further confirm that brassinin does not release H_2_S naturally, even in the presence of such agents. Negative controls—assessing H_2_S release from the aforementioned agents alone (without brassinin)—also produced no precipitate, as expected. The only detectable H_2_S generation was observed with 300 μM NaHS, and its H_2_S release remained consistent regardless of brassinin was present (Figure 4B).

### 2.5. Brassinin Induces CSE Expression and H_2_S Synthesis in SMCs

**Figure 5:**
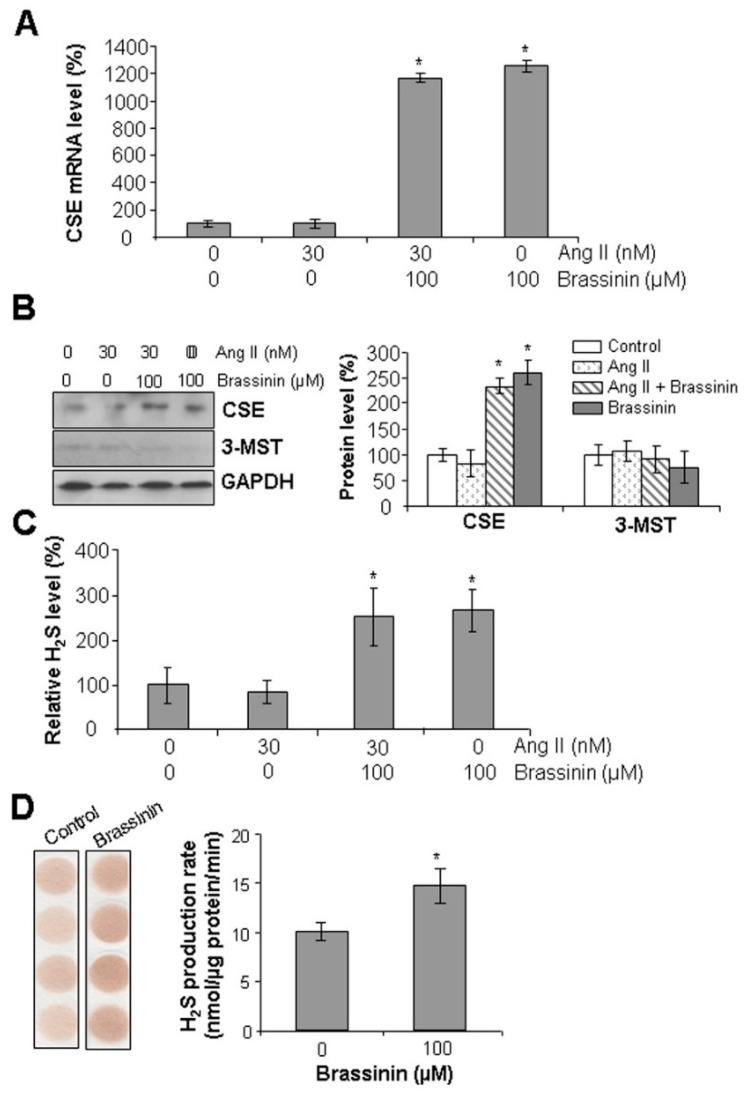
Brassinin induces CSE expression and H_2_S release in SMCs. After SMCs were incubated with 30 nM Ang II in the presence or absence of 100 µM brassinin for 24 h, CSE mRNA levels in cells were measured using real-time PCR (**A**), the protein levels of CSE and 3-MST were assessed with Western blotting (**B**), cells were stained with an H_2_S fluorescent dye WSP-1 (**C**) or H_2_S release was detected at 37 °C for 2 h with lead acetate assay in the presence of 10 mM L-cysteine and 2 mM P5P (**D**). n = 3 for (**A**–**C**). n = 4 for (**D**) *, *p* < 0.05 versus the control.

CSE is a major H_2_S-generating enzyme in vascular tissues [7]. To investigate whether brassinin modulates CSE expression in SMCs, we first analyzed CSE mRNA levels using real-time PCR. As shown in Figure 5A, SMCs were treated with or without Ang II and brassinin to assess CSE transcriptional activity. Compared to control cells, treatment with 30 nM Ang II did not significantly alter CSE gene expression. In contrast, supplementation with 100 μM brassinin markedly induced CSE mRNA levels. To validate these transcriptional findings at the protein level, we performed Western blot analysis. Consistent with the real-time PCR results, 100 μM brassinin significantly upregulated CSE protein expression compared to the control group (Figure 5B). The protein level of 3-MST, another H_2_S-generatin enzyme, was not altered by either Ang II or brassinin. Given these observations, we next evaluated brassinin’s ability to promote H_2_S production in SMCs using the H_2_S-specific fluorescent probe WSP1. As shown in Figure 5C, SMCs treated with 100 μM brassinin exhibited a significant increase in intracellular H_2_S levels. This was further corroborated by lead acetate paper assays (Figure 5D), demonstrating that brassinin treatment enhanced the H_2_S production rate in SMCs by 37.5%. Collectively, these data indicate that brassinin induces CSE expression and subsequent H_2_S release in SMCs.

### 2.6. Brassinin Stimulates C/EBPβ Binding to CSE Gene Promoter

**Figure 6:**
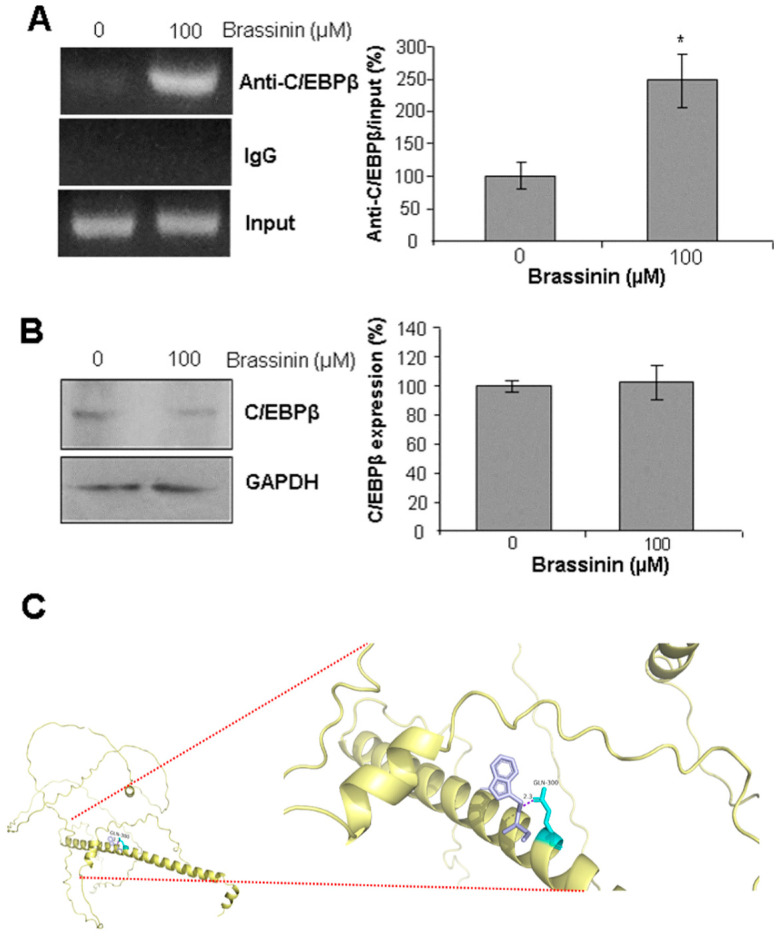
Brassinin stimulates transcription factor C/EBPβ binding to the CSE gene promoter. (**A**), Brassinin induced the binding intensity of C/EBPβ with the CSE gene promoter. After SMCs were incubated with 100 µM brassinin for 24 h, the cells were subjected to ChIP assay of C/EBPβ binding with CSE gene promoter. n = 3. *, *p* < 0.05. (**B**), Brassinin had no effect on C/EBPβ protein expression. After SMCs were incubated with 100 µM brassinin for 24 h, the cells were subjected to Western blotting analysis of C/EBPβ protein level. n = 3. (**C**), Molecular docking study showed that brassinin may form a stable hydrogen bond (2.3 kcal/mol) with glutamine 300 (GLN300), an amino acid residue located in the C-terminal region of C/EBPβ. C/EBPβ and brassinin were denoted in yellow and purple, respectively, and the blue area represents a hydrogen bond.

CCAAT/enhancer-binding protein beta (C/EBPβ) is a key transcription factor that regulates SMC functions and behaviors, and it also drives CSE transcription [24]. To further explore whether C/EBPβ is involved in brassinin-mediated upregulation of CSE mRNA levels, we performed ChIP assays to analyze the interaction between C/EBPβ and the CSE promoter. As shown in Figure 6A, brassinin significantly enhanced the binding of C/EBPβ to the CSE promoter compared to the control group. Notably, brassinin had no effect on C/EBPβ protein expression (Figure 6B), whereas molecular docking studies revealed that brassinin can form a stable hydrogen bond interaction (2.3 kcal/mol) with glutamine 300 (GLN300), an amino acid residue located in the C-terminal region of C/EBPβ (Figure 6C). These findings suggest that brassinin may bind to C/EBPβ, thereby increasing its binding affinity for the CCAAT box within the CSE promoter region.

## 3. Discussion

Increased dietary consumption of cruciferous vegetables offers a multitude of health benefits, with one key contributor being their production of sulfur-containing natural products [15,16]. Among these bioactive compounds, brassinin has been extensively validated for its antiproliferative and cancer chemopreventive activities across diverse experimental tumor models [21,25,26,27]. Additionally, brassinin exhibits antibacterial and antifungal properties [28]. However, there remains a paucity of information regarding its potential beneficial effects on vascular health. H_2_S is well established as a key mediator of cardioprotective functions, and numerous sulfur-containing natural compounds exert cardiovascular benefits through H_2_S release [9,11]. Against this backdrop, the present study aimed to investigate the regulatory roles of brassinin in SMC functions and evaluate its capacity to release H_2_S under varying conditions.

Ang II is a key peptide endocrine hormone involved in vasoconstriction [4]. In response to various stressors, excessive Ang II production can occur, leading to prolonged vasoconstriction and subsequent induction of vascular remodeling [5]. The present study confirmed that Ang II supplementation stimulated SMC proliferation and migration while inhibiting the expression of two smooth muscle contraction markers—key events in SMC phenotypic switching and the pathogenesis of various vascular diseases [2]. Brassinin is widely recognized for inducing cell cycle arrest and downregulating cell growth in diverse cancer cells [21,26]. Our findings provide evidence that brassinin at 100 μM had no effect on basal SMC proliferation or migration but effectively protected against Ang II-induced SMC dysfunctions. At this same concentration (100 μM), brassinin exerts no cytotoxic effects on several other healthy cell types, including adipocytes and macrophages, whereas higher doses exerted strong inhibitory effects on SMC proliferation [22,29]. Consistent with its ability to upregulate the expression of SM α-actin and calponin, brassinin also significantly increased the expression of myosin heavy chain in C2C12 myotubes—a critical protein for muscle contraction [30]. These results suggest that a low-to-moderate dose of brassinin is essential for maintaining normal muscle functions.

Increased oxidative stress and inflammation are frequently linked to SMC dysfunctions and vascular remodeling [1,2]. The present study confirmed that Ang II induced elevated ROS levels and upregulated inflammatory gene expression in SMCs, and these effects were significantly reversed by co-incubation with brassinin. The regulatory roles of brassinin in antioxidant and anti-inflammatory signaling pathways have been extensively documented. In cell-free and in vitro free radical scavenging assays, brassinin effectively neutralized superoxide and peroxyl radicals [23]. In cultured mammalian cells, brassinin mitigated oxidative stress by activating Nrf2-HO1-NQO1 pathway and suppressing the nuclear translocation of NF-κB [22,29]. Collectively, these findings strongly indicate that brassinin holds considerable potential for preventing the development and progression of CVDs.

We further investigated the molecular mechanisms underlying brassinin’s ability to reverse the detrimental effects of Ang II. Ang II primarily exerts its effects by binding to AT1R on SMCs, triggering vasoconstriction and thereby regulating blood pressure [5]. Ang II can also bind to AT2R, which mediates opposing effects to AT1R, including vasodilation [6]. Our results showed that Ang II upregulated the expression of both AT1R and AT2R, whereas brassinin specifically inhibited AT1R but had no significant effect on AT2R. These findings suggest that AT1R may be the key mediator through which brassinin exerts its protective effects against Ang II-induced SMC dysfunctions. To clarify how brassinin regulates AT1R expression, we further examined the impact of brassinin on ACE and ACE2—two critical enzymes involved in Ang II metabolism [4]. Notably, brassinin had no effect on the expression of either enzyme, excluding the possibility that brassinin modulates AT1R via changes in Ang II levels.

Brassinin contains two sulfur atoms covalently linked to the same carbon, forming relatively weak carbon-sulfur bonds [31]. Given the inherent instability of carbon-sulfur bonds compared to carbon-carbon bonds, which could be attributed primarily to differences in atomic size and orbital overlap between carbon and sulfur [31], it has been hypothesized that these sulfur-containing moieties might readily dissociate under certain conditions, potentially enabling brassinin to act as an H_2_S-releasing donor. To test this, we evaluated brassinin’s intrinsic H_2_S-releasing potential under various conditions using the lead acetate paper method. Unexpectedly, no H_2_S release was detected when brassinin was tested alone or when mixed with several sulfur-containing amino acids (L-cysteine, NAC, and GSH). This contrasts with many other natural sulfur-containing compounds, such as isothiocyanates and diallyldisulfide, which have been shown to release H_2_S in mammalian cells via non-enzymatic reactions with L-cysteine or GSH [11,32]. Additionally, although iron has been reported to interact with L-cysteine in the presence of P5P to induce H_2_S release [9], co-incubation of brassinin with both iron and P5P also failed to produce detectable H_2_S. Even the addition of an oxidizing agent (H_2_O_2_) did not facilitate cleavage of the carbon-sulfur bonds to generate H_2_S. Collectively, these results indicate that brassinin does not release H_2_S under any of the tested conditions. It should be noted that, while the lead acetate assay can specifically detect the free gaseous form of H_2_S (distinct from other sulfide species), its detection limit is ≥ 10 μM. Consequently, the lead acetate assay used in the present study may fail to detect H_2_S signals if the H_2_S released by brassinin is below this threshold. For more precise measurements, techniques such as gas chromatography coupled to mass spectrometry and HPLC would enable accurate quantification of H_2_S levels, with a detection limit in the nanomolar (nM) range [10,12].

This study further investigated the potential role of brassinin in inducing endogenous H_2_S generation within a biologically relevant context, using cultured SMCs. Real-time PCR and Western blotting analyses revealed that brassinin upregulated both the mRNA and protein expression of CSE—a key enzyme in endogenous H_2_S production [9,24]. This upregulation was accompanied by increased intracellular H_2_S levels, as confirmed by both the lead acetate paper assay and the fluorescent H_2_S probe WSP1. Unlike the detection of H_2_S release from brassinin, upregulated CSE protein—when supplied with its substrate (L-cysteine) and cofactor (P5P)—can continuously generate H_2_S over a 2 h assay period. This sustained H_2_S production is detectable even with the low-sensitivity lead acetate paper method. Similarly, after 24 h incubation with brassinin, SMCs accumulate high levels of CSE-derived intracellular H_2_S; this accumulation can be detected using WSP1, a highly specific H_2_S probe. Brassinin’s indirect enhancement of sulfur metabolism in SMCs aligns with previous reports on other sulfur-containing compounds, such as sulforaphane, which modulate H_2_S release by targeting sulfur-metabolizing enzymes [33].

Brassinin is recognized as a “multitopic” molecule, capable of acting as both a hydrogen bond donor and acceptor, which facilitates its interactions with various biomolecules. For instance, docking simulations and binding residue analyses have shown that brassinin inhibited tyrosinase by binding to its active site [23]. Brassinin also directly binds to GPX4 and NRF2, disrupting the interaction between COX-2 and the STAT3 promoter, thereby downregulating STAT3 activation [27]. Further mechanistic studies, incorporating both molecular docking analysis and ChIP assays, suggested that brassinin may interact with the transcription factor C/EBPβ and promote its binding to the CSE promoter, thereby driving CSE transcription. It should be noted that this evidence remains indirect and is associated with several limitations. First, due to the lack of available data on the three-dimensional structure of C/EBPβ’s N-terminal region, our docking analysis was restricted to the interaction between brassinin and C/EBPβ’s C-terminal region. Thus, we cannot rule out the possibility that brassinin may also interact with C/EBPβ’s N-terminal region. Additionally, further validation—such as redocking a known ligand to C/EBPβ’s C-terminal region—would help confirm the reliability of this docking prediction. Second, a luciferase reporter assay, which involves site-directed mutagenesis of the C/EBPβ binding region in the CSE promoter and is conducted in the presence of either wild-type C/EBPβ or C/EBPβ harboring the GLN300 point mutation, could yield more direct evidence. Despite these limitations, our findings still provide new insights into the molecular mechanisms underlying brassinin’s stimulatory effect on H_2_S signaling, supporting its potential as a CSE activator and a promising candidate for the prevention or treatment of CVDs.

**Figure 7:**
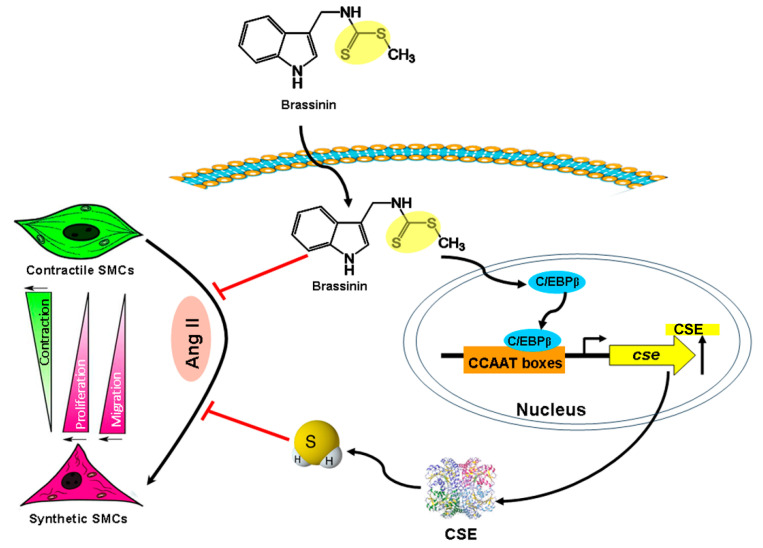
The proposed mechanisms by which brassinin induces H_2_S signaling and protects against Ang II-induced SMC phenotype changes. The diagram was created using PowerPoint 2021 (Microsoft).

Taken together, this study demonstrates that brassinin exhibits potent activity in inhibiting Ang II-induced SMC phenotypic switching, mediated through its anti-proliferative, anti-migratory, anti-oxidative, and anti-inflammatory properties. Although brassinin does not naturally release H_2_S under the tested conditions, it can stimulate endogenous H_2_S synthesis in SMCs by promoting C/EBPβ-mediated CSE transcription. This enhanced H_2_S production is likely to contribute to brassinin’s protective effects against Ang II-induced SMC dysfunction (Figure 7). These results strongly support brassinin’s beneficial role in improving vascular health. Further translational studies are warranted to validate its cardioprotective effects, utilizing angiotensin II (Ang II)-treated animal models followed by clinical testing.

## 4. Materials and Methods

### 4.1. The Reagents

All reagents were purchased from Sigma-Aldrich (St. Louis, MO, USA) unless otherwise specified. Pure brassinin (LKT Laboratories Inc., St. Paul, MN, USA) was dissolved in dimethyl sulfoxide (Thermo Fisher Scientific, Toronto, ON, Canada) to prepare a 100 mM stock solution. For experimental use, further dilutions were made in Dulbecco’s phosphate-buffered saline (PBS).

### 4.2. Cell Culture

Vascular SMCs were isolated from the aortic tissues of 8-week-old male mice as described previously [34]. SMCs were cultured in Dulbecco’s modified Eagle medium (DMEM) supplemented with 10% heat-inactivated fetal bovine serum, 100 U/mL penicillin, and 100 μg/mL streptomycin, and maintained at 37 °C in a humidified atmosphere containing 5% CO_2_. Cell cultures were washed every 48–72 h with 1–2 mL PBS before fresh medium was added. All downstream experiments were performed when cells reached 80% confluence.

### 4.3. H_2_S Detection

H_2_S release from brassinin was measured using a lead acetate paper assay [35]. Briefly, 100 µL of PBS solutions containing brassinin, L-cysteine, NAC, GSH, P5P, FeCl_2_, H_2_O_2_, and/or NaHS (as appropriate) were added to 96-well plates. A strip of lead acetate paper was then placed over each well, and the samples were incubated in the dark at 37 °C for 2 h to allow sufficient time for H_2_S production. H_2_S reacts with lead acetate to form a visible precipitate, which was quantified. The intensity of darkening on the lead acetate paper was analyzed using Image J 1.43 software, and H_2_S release was expressed in arbitrary units (AU).

The effect of brassinin on endogenous H_2_S generation in cultured SMCs was evaluated using both a lead acetate paper assay and the H_2_S-specific fluorescent probe WSP1 (Cayman Chemical, Ann Arbor, MI, USA). For the lead acetate paper assay: following 24 h treatments with Ang II and/or brassinin, cells were harvested and lysed in cold PBS. A 100 µL reaction mixture containing 20 µL cell lysate, 5 mM L-cysteine, and 2 mM P5P in PBS was added to 96-well plates, and a lead acetate paper strip was placed over each well. The reaction was incubated in the dark at 37 °C for 2 h. The intensity of darkening on the lead acetate paper was quantified using Image J 1.43 software, and H_2_S release was calculated against a NaHS standard curve, expressed as nmoles of H_2_S per microgram of protein per minute. For the WSP1 fluorescent probe assay: cells were first washed twice with PBS; and then incubated with 10 μg/mL WSP1 at 37 °C for 30 min to allow the probe to enter cells and react with endogenous H_2_S— a reaction that releases a fluorescent moiety. Fluorescence signals were captured using an EVOS M5000 fluorescent microscope (Thermo Fisher Scientific, Toronto, ON, Canada). Fluorescent intensity was analyzed with Image J 1.43 software and expressed as a percentage relative to the control group.

### 4.4. Cell Proliferation

The effect of brassinin on SMC proliferation was evaluated using the 3-(4,5-dimethylthiazol-2-yl)-2,5-diphenyltetrazolium bromide (MTT) assay [36]. Following 24 h treatment with varying concentrations of Ang II (1–100 nM) and/or brassinin (100–400 µM), cells were incubated with MTT labeling reagent (5 mg/mL) for an additional 4 h at 37 °C. During this incubation, only viable cells convert MTT to formazan using the mitochondrial enzyme succinate dehydrogenase. The formazan crystals were solubilized in dimethyl sulfoxide, and absorbance was measured at 570 nm using a FLUOstar OPTIMA microplate spectrophotometer (BMG LABtech, Ortenberg, Germany). The viability of untreated control cells was defined as 100%.

### 4.5. Cell Migration

Brassinin’s effect on cell migration was assessed using a wound healing assay. Cultured SMCs were first maintained in serum-free medium to suppress cell proliferation, ensuring that observations focused specifically on migration. A linear scratch was created across the cell monolayer using a pipette tip, and the plates were then incubated at 37 °C in a 5% CO_2_ atmosphere for 24 h. Following incubation, cell migration was quantified by comparing images captured before and after the incubation period using an EVOS M5000 phase-contrast microscope (Thermo Fisher Scientific, Toronto, ON, Canada), with analysis performed using Image J 1.43 software (National Institutes of Health).

### 4.6. Oxidative Stress Detection

The antioxidant properties of brassinin in SMCs were also evaluated using the 2,7-dichlorodihydrofluorescein diacetate (DCFDA) dye (Thermo Fisher Scientific, Toronto, ON) [36]. This fluorescent probe was added to the cultured cells, which were then incubated in the dark for 30 min to allow sufficient time for the dye to react with intracellular ROS, ultimately generating the fluorescent product 2,7-dichlorofluorescein (DCF). Fluorescence was visualized using an EVOS M5000 fluorescent microscope (Thermo Fisher Scientific, Toronto, ON, Canada) and quantified using Image J 1.43 software (National Institutes of Health).

### 4.7. Real-Time PCR

Total RNA was extracted from cultured SMCs using TRIzol reagent [23]. Complementary DNA (cDNA) was synthesized using reverse transcriptase and random hexamer primers (Thermo Fisher Scientific, Toronto, ON, Canada). mRNA expression levels were quantified with a real-time PCR using SYBR Green PCR Master Mix on a CFX Real-Time PCR Detection System (Bio-Rad Laboratories, Mississauga, ON, Canada). Primer sequences are listed in Table 1. The PCR protocol was as follows: an initial denaturation step at 94 °C for 5 min; 35 amplification cycles consisting of denaturation at 94 °C for 20 s, annealing at 62 °C for 30 s, and extension at 72 °C for 30 s; and a final melting curve analysis (94 °C for 15 s, followed by a ramp from 60 °C to 94 °C in 0.5 °C increments, each held for 15 s). Relative gene expression was calculated using the 2^−ΔΔCT^ method, where ΔCT represents the difference between the threshold cycle (CT) of the target gene and that of the endogenous reference gene GAPDH.

### 4.8. Western Blotting

Proteins from different treatment groups were separated using 10% SDS-PAGE gels with the Mini-Protean II system, run at 120 V for 90 min. Following separation, proteins were transferred to a polyvinylidene fluoride membrane at 120 V for 2 h. The membrane was then blocked overnight at 4 °C in 3% non-fat skim milk dissolved in phosphate-buffered saline with Tween 20 (PBST). After blocking, the membrane was incubated with primary antibodies against target proteins for 2 h at room temperature. These included anti-CSE (1:2000 dilution, Abnova, Taipei), anti-SM α-actin (1:1000, Cell Signaling Technology, Danvers, MA, USA), anti-calponin (1:1000, Cell Signaling Technology, Danvers, MA, USA), anti-C/EBPβ (1:1000, Cell Signaling Technology, Danvers, MA, USA), and anti-GAPDH (1:2000 dilution, Santa Cruz Biotechnology, Santa Cruz, CA, USA). The membrane was subsequently washed three times for 5 min each in 3% non-fat skim milk. Peroxidase-conjugated secondary antibodies (1:5000 dilution, Sigma-Aldrich, St. Louis, MO, USA) were then added, and the membrane was incubated for 2 h at room temperature. This was followed by three washes in 3% non-fat skim milk (as described above) and an additional three washes in PBST. Protein bands were visualized using enhanced chemiluminescence (ECL) substrate (Thermo Fisher Scientific, Toronto, ON, Canada). Densitometric analysis of protein bands was performed using Image J 1.43 software, with values normalized to the intensity of GAPDH (loading control).

### 4.9. Chromatin Immunoprecipitation (ChIP)

ChIP assays were performed to precipitate DNA fragments bound by the C/EBPβ transcription factor within the CSE promoter region [24]. Briefly, following 24 h treatment with brassinin, the cells were fixed with formaldehyde at 37 °C for 10 min and then sonicated in ChIP sonication buffer supplemented with protease inhibitors. After centrifugation, the supernatant was incubated overnight at 4 °C with rotation, using either an antibody against C/EBPβ or a nonspecific IgG antibody (as a negative control). A portion of the protein-DNA mixture was reserved without precipitation to serve as a positive control (input). The chromatin-antibody complexes and input samples were subjected to elution, cross-link reversal, and DNA purification. The C/EBPβ binding site within the CSE promoter was amplified using the following primers: forward 5′-GGAACGATCGGGGCAACACCT-3′ and reverse 5′-GCTGGGGCAGGAGTGCGAG-3′. The intensity of C/EBPβ’s binding to the CSE promoter was quantified using real-time PCR and normalized to the input level using the same primers.

### 4.10. Molecular Docking

The protein structure information of C/EBPβ was derived from the Protein Data Bank (https://www.uniprot.org/uniprotkb/P17676/entry, accessed on 1 July 2025). The 3D structure of brassinin was downloaded from the PubChem Compound database (https://pubchem.ncbi.nlm.nih.gov/, accessed on 1 July 2025). Molecular docking of brassinin with C/EBPβ was performed using AutoDock Vina software v1.1.2 (Scripps Research), with a focus on the experimentally validated X-ray diffraction data corresponding to the 250–340 amino acid region of the protein [23,27].

### 4.11. Statistical Analysis

Data are presented as means ± standard error of the means (SEMs) from at least three independent experiments. Statistical analyses were performed using Student’s *t*-test or one-way analysis of variance (ANOVA) followed by Tukey’s post hoc test, depending on the number of experimental groups. The data met the statistical assumptions as determined by SPSS Statistics 20.0 (IBM). A *p*-value < 0.05 was considered statistically significant.

## Figures and Tables

**Table 1:** Primers used for real-time PCR.

Primers	Sequences
CSE	Forward: 5′-ATCAGCACCCAGAGCCAAGG-3′ Reverse: 5′-AGCGATCACACCACAGACCAAG-3′
TNFα	Forward: 5′-CCCACACCGTCAGCCGATTT-3′ Reverse: 5′-GTCTAAGTACTTGGGCAGATT-3′
IL2	Forward: 5′-CGCAGAGGTCCAAGTTCATC-3′ Reverse: 5′-AACTCCCCAGGATGCTCAC-3′
IL4	Forward: 5′-CGAGCTCACTCTCTGTGGTG-3′ Reverse: 5′-TGAACGAGGTCACAGGAGAA-3′
IL6	Forward: 5′-ACCAGAGGAAATTTTCAATAGGC-3′ Reverse: 5′-TGGGAGTGGTATCCTCTGTGA-3′
ACE	Forward: 5′-ACCCCACCCTCTCGCTACAACTTC-3′ Reverse: 5′-GACTTCGCCATTCCGCTGATT-3′
ACE2	Forward: 5′- TGGCGCACGGGGAAAGA-3′ Reverse: 5′- GCTGAAGGGCCTGTAGTTGACG-3′
AT1R	Forward: 5′-CCCCGAGGCAAGCAGAATAGGTG-3′ Reverse: 5′-GCCCGTGAGAGCAGCTGGTTGAAG-3′
AT2R	Forward: 5′-AGTCCGCCTTTAATTGCTCACAC-3′ Reverse: 5′-CCAAACAAGGGGAACTACATAAGA-3′
GAPDH	Forward: 5′-TCTCCTGCGACTTCAACAGC-3′ Reverse: 5′-GGTGCACGAACTTTATTGATGGT-3′

## Data Availability

The original contributions presented in this study are included in the article. Further inquiries can be directed to the corresponding authors.
